# Different ways… same message? Road safety-targeted communication strategies in Spain over 62 years (1960–2021)

**DOI:** 10.1016/j.heliyon.2023.e18775

**Published:** 2023-08-01

**Authors:** Mireia Faus, Cesáreo Fernández, Francisco Alonso, Sergio A. Useche

**Affiliations:** a INTRAS (Research Institute on Traffic and Road Safety), University of Valencia, Valencia, Spain; b Department of Communication Sciences, University Jaume I. Castellón, Spain; c ITACA (Research in Technologies Applied to Audiovisual Communication) Research Group, Spain; d Faculty of Psychology, University of Valencia, Valencia, Spain

**Keywords:** communication and media, Advertising, Road users, Traffic safety, Spain

## Abstract

Among the most generalised preventive measures against traffic crashes, advertisements and broadcast campaigns in the media have stood out over the last six decades. The core aim of this paper is to describe the evolution of the subject matter and typology of road safety-related advertisements used in Spain during 62 years (1960–2021). Thus, this paper assesses their role in reducing road fatalities, while keeping in mind the potential effect of the many other road safety-related preventive measures carried out in the country during this period. The results of this study allow us to target five key time periods, all of them with clear particular communication strategies to be differentiated, using specific types of advertisements and informative, persuasive, emotional, and humorous techniques (among others) to reach the audience. Additionally, some key practical implications and guidelines are provided.

## 1 Introduction

The latest data indicate that 1.35 million people die each year due to road traffic crashes [1]. Although this figure is self-explicative, solving this public health concern has been proven to imply several continuous efforts from multiple stakeholders [2,3]. For this reason, government entities in different countries develop strategic road safety plans in which they establish the planning for actions, strategies, and measures to be adopted to prevent road crashes [4,5]. Therefore, a notable fact in this regard is that over the last decade, the number of traffic crash victims has been reduced in many –although not all– countries worldwide [6].

Preventive actions and measures have been aimed at improving vehicle conditions [7], improving infrastructures [8], and raising awareness in society [9]. This last point has been especially attended to due to recent studies endorsing the great influence of human factors on traffic crash rates [10,11]. In this regard, the most common theoretical budget of literature claims that improving road safety behaviours of a population (or a group within it) and promoting risk avoidance are actions commonly associated with decreases in the number of traffic casualties, given that their causal role as *crash inhibitors* remains scarcely supported by empirical evidence [12,13].

In this sense, while aiming to improve the matter's state of affairs, some actions have been developed in many countries across the last six decades. For instance, road safety education programs have been designed [14], further regulations have been developed, and enforcement strategies (i.e., law-compliance targeted punitive actions) have been applied. Moreover, many communicative developments, paradigms, and models aimed at raising awareness among road users have been implemented over time [15].

Precisely, communication campaigns have been fundamental for this purpose, generally acting as a complement to the rest of the measures adopted and as the “friendly” way to approach the public [16]. These campaigns, framed within the so-called social marketing, characteristically constitute informative and persuasive advertising strategies aimed at promoting attitudes and behaviour that are beneficial to society [17,18]. In this case, the literature supports the idea that the purpose of a typical traffic advertisement is to alert the population to the dangers of inappropriate driving, with the intention of reducing both road crashes and mortality rates [19].

In Spain, the main body in charge of designing and developing advertisements and awareness campaigns is the Directorate-General for Traffic (DGT). This organisation was born with the objective of developing actions aimed at training and improving the behaviour of road users, and the safety and fluidity of vehicle circulation [20,21]. Therefore, in addition to communication campaigns, it has been involved in strategic national road safety plans, from which actions have been carried out in the areas of education and training, regulations and their enforcement, vehicle safety, infrastructure and ITS, professional transport, victims of traffic crashes, and research and management, among others [22].

## 2 Literature review

### 2.1 Effectiveness of communication campaigns in modifying behaviour

The general aim of social advertisements includes educating and raising awareness in the population about a problem, in order to achieve a change in behaviour that will contribute to a better social wellbeing [23]. Therefore, communication campaigns of this type intend to be an accelerator in social change by spreading ideas so that social acceptance of such ideas increase, and educating about specific behaviour to achieve an increase in its practice.

On occasions, the heterogeneity of the social marketing discipline makes the presentation of complete and coherent theoretical models difficult [24]. In this way, some theoretical approaches are extrapolated from the developed models in this commercial marketing field, which provokes certain difficulties in the adaptation of said theoretical models.

In any case, a consensus exists between multiple authors that communication strategies are valuable in influencing an audience, since intervention tools and mechanisms about the spectators' behaviour are employed [25]. On one hand, social advertisements consists of an informative component, which provides data and relevant information for the spectator, which is susceptible of being acquired and remembered. On the other hand, they also consist of a persuasive component related to its power to convince the audience about the importance of exercising determined actions and behaviour [26]. In this sense, the mechanisms of persuasion can be rational, emotional, or even make use of one's unconsciousness. The rational strategies are centred around convincing the receiver through real objectives, powerful arguments and relevant information that can capture the attention of users. Likewise, emotional mechanisms play with individual's emotions and empathy, and exposes them to situations or people affected by the topic, so that they are made aware of this and decides to act accordingly. Finally, the strategies linked with the unconsciousness are marked in subliminal advertisements, and consist of convincing viewers without the focus of the advertisement being on the topic, but rather a secondary element.

The main variables to evaluate the effectiveness of social communication campaigns are its impact, memory, an increase in awareness and, above all, the audience's change in attitudes and behaviour. Nonetheless, it is important to highlight that there are a wide range of variables and factors that come into play in the evolution of an individual's behaviour, which means that isolating a spot in this process is complicated, especially when measures are taken to control external values [27]. For this reason, many authors prefer to use the term “contribute”. Therefore, a campaign is not the only factor in the change in attitudes and behaviour, but rather it contributes to said changes along with other factors that act positively, favouring the evolution, or negatively, making a change in habits difficult.

Additionally, there are several variables that scientific evidence associates with the effectiveness of social advertisements. Some refer to the campaign's characteristics and its format, such as the position of the advertisement, its repetition and/or frequency of emission, the duration of the spot, the publicity style, the tone of the message, and the communicative strategies used [28]. However, there are also variables that concern the characteristics of the spectator themselves, such as the implication of the audience in the issues tackled in the spot, attitudes towards publicity in general (and particularly social publicity), the level of plausibility of the spot, personal perception about the media of broadcast of the campaign, and about the broadcasting entity of the advertisement [29].

In any case, the existing knowledge about the effectiveness of social publicity is limited due to the lack of evaluation of these types of campaigns [30]; a phenomenon that may be related to the difficulty of measuring social marketing campaigns, and to the financial costs that this implies, among other factors. However, the measurement of effectiveness is fundamental to understand what variables, resources and/or strategies are most useful for a message to be memorable and perceived as beneficial to society, and to identify the degree to which an idea is integrated in a population, thus providing information about change in behaviour derived from the broadcasted communication campaigns [31].

### 2.2 Importance of communication campaigns in road safety: findings from literature

Traffic campaigns are a type of social campaign, and they are one of the main preventative measures that is applied in the road safety sector. Each year, tens of thousands of advertisements and audio-visual pieces, which are broadcast to the population, are developed by organisations and entities responsible around the world. Road safety campaigns primarily aim to persuade road users to follow the traffic laws and rules when driving, and emphasise precautions and the need to avoid risky road driving [32,33]. Therefore, advertisements and communication campaigns are aimed at both informing and educating citizens about safe driving and movement, as well as alerting them about the negative consequences of dangerous driving behaviour [34]. Thus, the type of communicative strategies and resources are heterogeneous, using realistic techniques with great emotional impacts, as well as more rational techniques that provide road users with relevant information.

Another relevant issue remarked in literature is related to the organisations responsible for the creation and dissemination of the aired communication campaigns, which require great knowledge in the population's communicative processes for whom the spot will be broadcast, as well as the characteristics and peculiarities of the target audience [35]. These elements will allow a spot to be designed that captures the attention of a determined audience and, at the same time, make it more likely that psychological barriers that may impede spectators from adequately receiving the message are broken. The acceptance of the persuasive message will, therefore, be determined by the degree of convincingness that they are able to achieve [36]. It is for this reason that the importance of studying the needs of the audience is emphasised, to be able to adapt the spot to its characteristics, as well as implementing emotional and impactful elements that activate an emotional response in the target audience.

In specific regards to the evaluation of the traffic campaigns aimed at changing behaviour, a scope review of literature has been carried out. On one hand, although not surprising given the precedents with social publicity, there are very few investigations or studies that aim at evaluating the effectiveness of traffic campaigns. On the hand, the majority of the examined evaluations used a non-experimental or observational cross-section methodology, using a variety of techniques such as surveys during and after the campaign's broadcast, structured interviews, and focus groups, and data from road crashes [32]. However, the use of experimental or almost-experimental methods, where the investigator manipulates relevant variables to verify if changes in behaviour occurred (or not), is rather infrequent.

In terms of the main findings from the scientific literature, there is no unanimous conclusion about the effectiveness of communication campaigns for changing behaviour. Some studies identify significant changes in the attitudes and behaviour derived from the visualisation of certain traffic announcements [37,38]. However, results from other studies, either do not show significant changes, or the changes are very slight [39,40].

In any case, most studies highlight the ability of the campaigns to increase the efficiency of other preventative measures. That is to say, presenting a spot as a complement to other actions, such as road awareness education, police controls, or sanctions, substantially increases the results in comparison to those that are produced applying such actions in an isolated manner [16,41]. Further, recent literature reviews stress the importance of analysing campaigns in the broadcasting context, and bearing in mind the rest of the preventative measures that have been developed at the same time, and thus the consequences of this joint or complementary application on the prevalence of risky behaviour [32].

### 2.3 The case of Spain: evolution of preventive measures and their relationship with traffic crash rates

**Fig. 1:**
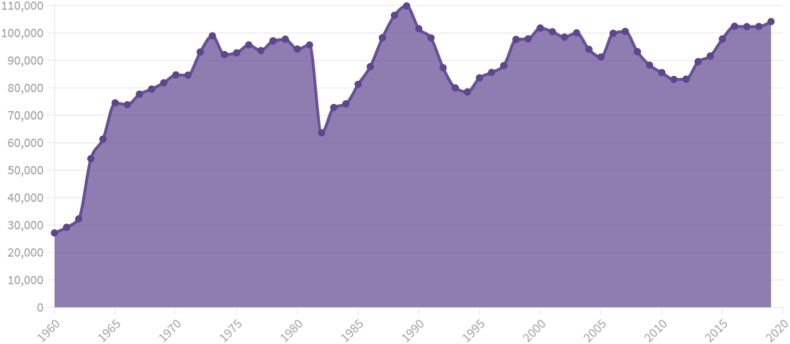
Number of traffic crashes with casualties registered in Spain (1960–2019).

At first glance, the evolution of traffic crash rates in Spain over the six decades addressed shows a clear relationship between the main milestones achieved by the DGT and road safety over the years. Since 1960 there has been a rapid increase in the number of road crashes, influenced by the constant growth of the driving population (Fig. 1). The first turning point occurs at the beginning of the 1980s, with a sharp drop in the number of crashes with casualty-related outcomes. This phenomenon is undoubtedly influenced by the approval of the nation's first National Road Safety Plan in 1979, which marked the actions and strategies of the following years [42]. These were years in which many of the features that are still used nowadays were introduced, such as the STOP sign (1979), alcohol controls (1981), compulsory use of helmets on motorcycles (1891–1982) and compulsory psycho-technical tests for all motor vehicle drivers (1985).

**Fig. 2:**
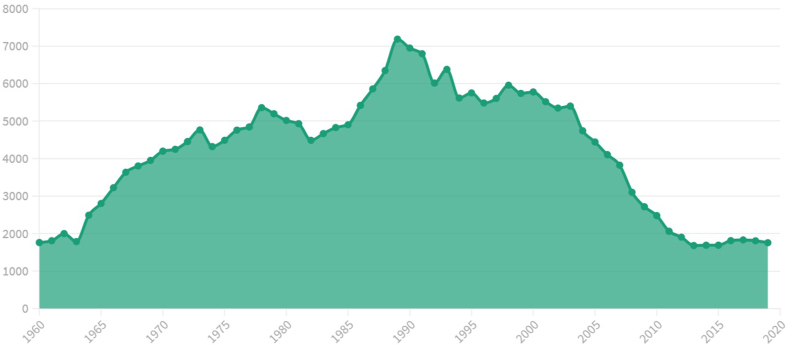
Number of deaths resulting from traffic crashes in Spain (1960–2019).

However, the decade of the 80s marked a relentless increase in the number of traffic crashes again, reaching the highest figure of the 20th century in 1989 with 109,804 events, which resulted in 9344 deaths, 52,418 seriously injured and 116,993 slightly injured (Fig. 2). At that time, a rethinking of this problem by the authorities was necessary, and consequently, the Traffic, Motor Vehicle Circulation and Road Safety Law was passed. This meant an increase in investment in publicity campaigns and the establishment of Road Safety Education as a transversal axis for traffic crash prevention [43].

In the following years, the downward trend in traffic crash rates continued. This coincided with the establishment of new regulations, such as mandatory seat belts in cities (1992), mandatory child restraint devices (1992), a distance of 1.5 m for overtaking bicycles (1992) and the reform of the penal code (1993). Until 1995, there was an upturn in the data and then a certain stagnation. During these years, there were multiple reforms in the rules and regulations for drivers and vehicles, as well as the enactment of important measures, such as the new blood alcohol levels (1999), the Rosita project for the detection of drugs at the wheel (2002) or the compulsory wearing of helmets for cyclists on the road (2004).

In 2005, the Strategic Road Safety Plan 2005–2008 was drawn up, and the Road Safety Commission was created in the Congress of Deputies and, for the first time, the Report of the State Attorney General's Office dealt with traffic offences [44]. In addition, a law regulating the Points Permit was passed, which was to be implemented on July 1 of the following year (2006) and marked the beginning of a new period of constant road crash reduction in Spain. From that moment on, all new cars should have seat belts for all their seats, something that would also occur for new buses (2007).

In 2010, the Strategic Road Safety Plan 2011–2020 was drawn up, with which a historical low of victims was reached, with 1126 fatalities; 4843 seriously injured and 1018 fatal events in 2015 [45], as graphically shown in Fig. 2. Furthermore, in 2016, Road Safety offences fell to 23.8% of the total number of offences (these accounted for 44% in 2008). Additionally, several measures were developed to protect cyclists, through safe routes and special surveillance plans for this vulnerable group during these years.

All the measures described have been complemented with other actions, specifically related to communication and awareness campaigns, which have contributed to the education and spreading of information across the population. The present investigation is centred around analysing the characteristics of advertisements emitted by the DGT over the years and examines their contribution in the reduction of deaths caused by road traffic crashes.

### 2.4 Research objectives

In this sense, the aim of this study is to review the historical background of communication campaigns in the traffic and road safety sector that have been broadcasted in Spain. In particular, we will explore the evolution in relation to the content and the communicative strategies of the advertisements, in order to determine different stages or time periods, whose spots present similar characteristics. Additionally, preliminary conclusions will be extracted on the effectiveness of the communication campaigns, through the comparison with the crash and fatality rates in Spain. In this sense, their complementary role to other preventive measures applied in the country will be identified. However, in no case will a direct correlation be established between the traffic ads and the data, since it is impossible to completely isolate the specific effects of the campaigns from the rest of the factors and preventive actions applied at the same time.

## 3 Methods

### 3.1 Description of the methodology and process of content analysis of the campaigns

The present study provides a first approach to analyse the effectiveness of communication campaigns in the traffic and road safety sector in Spain, given the scarcity of existing evaluations. For this purpose, an exhaustive review and analysis of the content will be carried out to identify the communication strategies used in different time periods.

In this sense, the process developed in this research consisted of seven steps described below.

1) Defining the objectives of the analysis: initially, the main objective of the content analysis is established, which refers to the identification of trends or changes in the communication strategies used in the spots over time. As a hypothesis, an evolution and change in the techniques and communicative resources is expected, as well as in the content of the campaigns, allowing for the identification of time periods in which campaigns present similar characteristics.
2) Establishing selection criteria: it is impossible to collect all the campaigns broadcast in Spain for different reasons related to availability, access to information and the volume of advertisements, among other factors. Therefore, specific inclusion and exclusion criteria have been determined for the communication campaigns to be analysed, in order to make the study systematic and replicable. The criteria are set out in section 3.2. And were agreed upon by the authors of the manuscript.
3) Collecting the communication campaigns: once the search and selection criteria were established, the audiovisual pieces to be analysed were collected and extracted.
4) Viewing and recording the campaigns: the traffic ads were viewed independently by the authors of the manuscript. Each campaign was individually rated on the basis of their attributed dimensional significance (percentage), keeping those spots with an average of≥ 75%. Information about the content of selected campaigns was recorded in a data table that included the variables described in section 3.3. Thus, the authors acted as independent reviewers, each carrying out the process of identifying the main categories or components of the ads. Any discrepancies that arose were resolved by means of consensual decisions.
5) Identification of stages or time periods and qualitative analysis: the characteristics of the ads allowed us to group them into different time periods. Each stage presents a set of spots broadcast at specific times and with similar characteristics in relation to their content and the communicative strategies used. A detailed description has been made of the particularities of each of the stages identified in this research. Additionally, a specific name has been designated for each stage based on its unique and differential characteristics, as well as on previous literature [46,47].
6) Quantitative analysis: descriptive and frequency analysis have been conducted with the recorded data. Firstly, the evolution of the communicative resources used in the established stages has been identified numerically, making it possible to point out potential differences and determining the trends present in each period. Secondly, a content analysis of the campaigns' slogans has been carried out, identifying the most repeated words and messages in each time period.
7) Preliminary study on the effectiveness of the campaigns: it is not possible to analyse the effectiveness of the campaigns in isolation because their application as a preventive measure on the population was not independent of other actions developed simultaneously. However, it is possible to analyse the role of the communication campaigns as a complement to other measures, such as police reports and statistical crash records, with this being the usual way of evaluating the effectiveness of different preventive measures in this sector [48].

### 3.2 Determining the Study's sample of communication campaigns

The Directorate-General for Traffic (DGT) has developed and implemented hundreds of communication campaigns over the past years. Therefore, it would indeed be impossible to carry out an exhaustive analysis of all of them, given the large number of advertisements, documents and records widespread over all the existing archives. Therefore, a practical solution was raised by an expert group: fixed criteria to obtain and select a reasonably high number of communication campaigns filling up a set of requirements were established, as detailed below.

1) Thematic. The main theme of the chosen spots is road safety, mobility and/or traffic, and may cover different subjects within this field. Thus, the covered elements may be risk factors in crashes, crash prevention measures, vulnerable users, traffic regulations and laws, sustainable mobility or attention to victims, among others.
2) Issuing entity. The campaigns must have been developed by the DGT, thus no spots that were developed by other companies or entities were selected.
3) Multiplatform. Advertisements that were only broadcast through radio spots or street posters were not included. The communication campaigns selected include an audiovisual piece broadcast on television and at least one other means of communication with users (radio, press, social networks or other media).
4) Availability. The spots must be available for viewing and analysis, something that does not happen in many cases. All the selected campaigns have been obtained from the DGT database and from video websites, in particular YouTube, Vimeo, and other audio-visual repositories. This does not mean that the campaigns were necessarily broadcasted on these video platforms to the audience; instead, these are the outlets in which these spots are still available for viewing. This way, and even though these campaigns are currently discontinued, they are still accessible for analysis and description of their contents (see Appendix I).
5) Temporal duration. Communication campaigns that have been exposed to the public for a prolonged period of time have been selected. Therefore, we have not taken into account specific spots or campaigns linked to specific events that have been broadcast for less than two weeks.
6) Scope. All the communication campaigns selected were broadcast at a national level. Traffic campaigns carried out jointly with Spanish Autonomous Communities or regions that had a local scope have been discarded.

After excluding traffic ads that did not meet all these requirements, a sample of 104 communication campaigns broadcast from 1960 to 2021 was obtained.

### 3.3 Coding the variables

After collecting the pertinent information, a careful analysis of the content of the selected spots was carried out. The objective of this was to characterise and understand the evolution of traffic communication campaigns in Spain. In other words, we looked to identify common trends and patterns among the advertisements, allowing us to differentiate with a certain level of clarity the stages that happened in terms of the development of road safety-related spots over the 62 years analysed.

In this sense, tables will be drawn up showing the following variables.

1) Campaign and/or its main slogan: The main message of the campaign is presented and, if it exists, its slogan. This information allows us to analyse the evolution of the content and the communicative intention of the DGT messages, as well as to evaluate the frequency or repetition of certain words or concepts according to the time period in which the spots were designed and broadcast in Spain.
2) Year of broadcast: The year of broadcast of each advertisement is indicated, which allows us to locate the contents and the communicative strategies used at a certain point in time.
3) Theme: the main theme addressed in the campaigns is identified in relation to the risk factor and/or risk group involved. In this sense, the subject matter may be related to speeding, alcohol and/or drug consumption, distractions, helmet use, telephone use, vehicle maintenance, pedestrians, cyclists, children, seat belt use, emergency situations, commuting trips, and/or generic holiday travel advertisements, among others.
4) Marketing technique or resources used in the spot: After viewing the spot, the communicative resource used is identified through a consensus among the authors of this manuscript. In this way, a main marketing technique is assigned to determine the tone and intentionality of the audio-visual piece. Specifically, the spots may be:
  a. Informative: the message is presented in an exclusively descriptive and explanatory way, with the intention of educating the viewer, but without transmitting any type of emotion.
  b. Animation: cartoons and/or other types of animations are used to convey the message.
  c. News: the campaign presents the viewer with real news about crash rates, about a specific causalty, or about any other aspect related to road safety.
  d. Graphics: graphics on crash rates of various types are presented to alert the viewer about the importance of the problem addressed.
  e. Personalities: the star-system resource is used, in which a famous or relevant person in the country is in charge of spreading the message.
  f. Metaphorical: the message is not transmitted to the viewer in a literal way, but metaphors, rhetorical figures or symbolic images are used to generate a parallelism between the scene described and the risk factor addressed in the spot or its consequences.
  g. Impact: images of high emotional impact are used, through the use of scenes with a high level of realism, violence and/or aggression. Generally, the degree of rawness in the images and the message is intended to generate fear in the viewer to prevent them from reproducing risky behaviours.
  h. Emotional: the content of the advertisement aims to generate emotions without using violent images. In particular, they intend to generate empathy in the viewer through testimonies from victims or family members, direct messages to the user and reflective messages.
  i. Comparative: different situations are presented in the context of traffic to be compared with each other, especially showing the differences between the consequences of complying with the rules in a given situation and the consequences of violating them by engaging in risky behaviour.
  j. Humorous: the main resource of the campaign is humour. This category includes different types of humorous resources, from the presentation of a monologue by a comedian on traffic situations, to the presentation of black humour and/or sarcastic scenes to generate reflection.
5) Descriptions of the content of the advertisement (available in Appendix I). Each selected campaign has been viewed, and its content described in detail, in order to contextualize the findings obtained.

## 4 Results and discussion

In order to present a successive analysis and theoretical/empirical discussion of the key results of the data extraction carried out in this study, this section is divided in three subsections: evolution of (general) communication campaigns; evolution of (traffic-related) campaign; and the contribution of traffic announcements to the evolution of road mortality rates, exploring their role as a complementary measure to other preventive actions.

### 4.1 Evolution of general communication campaigns in Spain

The evolution of the advertising campaigns carried out by the DGT in Spain can be observed both in the topics that they deal with and the way in which they are approached (DGT, 2021b). Since the beginning of the 1960s, a variety of techniques and tools have been used to raise awareness among the population. The first step in understanding the changes that have taken place over the years in the communication campaigns is to find common patterns in terms of themes and communication techniques used. Thus, after examining all the campaigns developed by the DGT and the few studies that have been carried out to date (Segura-García, 2015; Villajos, 2008), five time periods have been established. These five time periods present campaigns with particular characteristics in terms of content, themes, and marketing techniques.

#### 4.1.1 The beginnings (1960–1978)

The first stage covers the years 1960–1978. Table 1 summarises the characteristics of the advertisements of this period. This was a historical moment when the use of vehicles was restricted to a very specific sector of the population. This sector was made up mainly of middle-aged men who used them for commuting to work and during vacation periods. For this reason, the target audience for most of the advertisements was the male private car driver. Among the topics covered were vehicle maintenance, overtaking, speeding, alcohol consumption while driving and taking care of vulnerable users. In addition, in the mid-1960s, a campaign aimed at pedestrians, especially children, was carried out for several years, promoting safe travel and road safety education at home.

**Table 1:** Characteristics (key features) of national traffic campaigns between 1960 and 1978.

Campaign	Year	Theme	Technique
Respect the signs	1960	Signs	News
One drink too many can be one life too short	1961	Alcohol	Animation
Overtaking is dangerous	1962	Overtaking	Animation
Drive at a safe speed	1962	Speeding	Animation
Look first, then cross	1963	Crossing	Informative
Always cross with a green light	1964	Pedestrian	Animation
Wear … Helmets	1964	Helmet	Animation
Attention to speed	1965	Speeding	Informative
Cyclists on the right one after the other	1965	Cyclists	Informative
Cycle on the right	1965	Commuting	Informative
Check your lights	1965	Vehicle maintenance	Informative
Check your lights	1967	Vehicle maintenance	Informative
Weaker ones need more protection	1967	Children	Informative
The weaker need more protection	1967	Children	Animation
Don't be like him	1969	Speeding	Informative
Teach him to ride well	1969	Pedestrian	Animation
Pedestrian! Always keep to the left side of the road!	1973	Pedestrian	Animation
Pedestrians also have their rights	1973	Pedestrian	Animation
Knowing how to walk is not enough; teach him how to drive	1973	Pedestrian	Informative
Driving at night	1973	Pedestrian	Animation
For your safety, use seat belts	1973	Seat belt	Animation
Give them time	1976	Pedestrian	Informative
Always with one glass less	1978	Alcohol	Animation
When in doubt, don't go ahead	1978	Overtaking	Informative

Since the population was found in the initial stages of familiarisation with the rules of circulation, the transmitted messages were basic and simple in their nature. The teaching of road behaviour was carried out in a demonstrative way, that is to say, through the representation of situations in which people or vehicles demonstrated appropriate behaviour, providing the spectator with instructions on how to act. The transmission of information was largely based around an informative focus, using animations as well as people and real situation. Similarly, in the first advertisements, repetition was used as a persuasive strategy. In them, the voiceover pronounced phrases such as “respect the signs” (1960) or “wear a helmet” (1964) repeatedly over images of vehicles on the road. Moreover, in most cases, they are isolated advertisements and not so much a set of audio-visual pieces included in the same communication campaign.

#### 4.1.2 The ‘soft’ line (1979–1991)

At the end of the 1970s, a change in message is observed. Table 2 shows the campaigns of this period in detail. The behaviour that road users should perform is no longer exposed from this moment on, but rather the negative consequences of not making safe journeys are shown. In this way, special emphasis is placed on travel by private car, without neglecting the broadcast of specific advertisements on risk factors such as alcohol, seat belts or helmets. These are the beginnings of audio-visual pieces that focus on the relevance of strengthening road safety through the promotion of responsible behaviour, seeking to raise awareness of the importance of crash prevention or, in the case that this did occur, establishing guidelines to mitigate their consequences of possible victims. There is a minimum emotional component aimed at highlighting that life can be lost on the road and the responsibility of users in crash prevention. Thus, slogans such as “Holidays are for living” (1983), “You are the most important part” (1974) or “Many lives depend on you” (1987) appear.

**Table 2:** Characteristics (key features) of national traffic campaigns between 1979 and 1991.

Campaign	Year	Theme	Technique
Helmet, the only mandatory garment	1981	Helmet	News
Keep an eye on your tires	1981	Vehicle maintenance	Graphics
City in chaos	1982	Travel	Graphics
Vacations are for living	1983	Travel	Informative
Thank you for driving well	1984	Travel	Informative
Be prepared for winter driving	1984	Weather conditions	Informative
You are the most important part	1984	Travel	Informative
If you drink, don't drive	1985	Alcohol	Personalities
In every car there are human lives	1985	Travel	News
In a car, you are the most fragile thing	1986	Travel	Informative
Many lives depend on you	1987	Travel	News
Use the best car insurance is caution	1988	Alcohol	News
On the road, don't go out like bullets	1988	Belt	Informative
A friend tells you	1989	Belt, alcohol, helmet, speed	Graphics
Pedestrian, there is nothing as fragile as your body	1990	Vulnerable users	Graphics
Don't make your trip an adventure	1990	Travel	Personalities
A champion's word. Don't take risks. You'll come out on top	1991	Travel	Personalities
Get it on your head	1991	Helmet	Metaphorical
Buckle up	1991	Belt	Informative

In any case, the most used resource are still real and animated images, without showing aggressive scenes or with a certain visual impact. A typology of ads with a metaphorical component is introduced, as well as the “star-system” resource, consisting of the appearance of personalities in the ads. The aim is to check whether a figure with social relevance could have an impact on the change of behaviour of the population. This resource can be found in the campaign “Don't make your trip an adventure” (1990) with Harrison Ford as Indiana Jones, in “A champion's word. Don't take risks. You'll come out on top” with Carlos Sainz (1991), or in the well-remembered “If you drink, don't drive” ad (1985) starring Stevie Wonder.

In this period, it is worth mentioning the campaign “A friend tells you” (1989), which consists of an animation of a squirrel superimposed on real scenes. In this campaign, the squirrel interacts with drivers, teaching them how to behave in potentially dangerous situations. It is the first campaign to include the same slogan in different visual pieces that are cohesive, thus transmitting a more global message to the viewer.

The year 1989 marks a turning point for the DGT, because it sees the highest number of fatalities in a year up to that time. Subsequently, it would also become the highest number of deaths in traffic crashes in a year in the history of Spain. However, from that moment on, this numbers starts to decrease, as shown in Fig. 2.

#### 4.1.3 The ‘hard’ line (1992–1997)

The so-called *hard-line* was developed during practically the entire decade of the 90s, and is characterised by the introduction of highly emotional and impactful components in its ads, as detailed in Table 3. At this stage, it is already common to find several audio-visual pieces with a common component that encompasses them, and where each advertisement focuses on a different risk factor. In this sense, seat belts, helmets, alcohol consumption, speeding and fatigue are the most frequently reproduced. Furthermore, the profile of the protagonists of the spots is more varied, with children, young people and adult drivers appearing, among others. This is a time when driving is completely normalised for all types of people, and advertisements must adapt to this reality in order to make all these user profiles feel identified with and, consequently, raise awareness in society as a whole.

**Table 3:** Characteristics of national traffic campaigns between 1992 and 1997.

Campaign	Year	Theme	Technique
You pay for your recklessness more and more	1992	Helmet, seat belt, speeding	Impact
In the end, recklessness is paid for	1993	Helmet, seat belt, speeding	Emotional
The story of …	1994	Helmet, signs, alcohol, seat belt, speeding.	Emotional
You're not the only one who pays for your recklessness	1995	Safety distance, fatigue	Emotional
The solution is in your hands	1996	Alcohol, seat belt, speeding, fatigue	Emotional
Show your friendship, do not allow recklessness	1997	Alcohol, seat belt, speeding	Impact

During this period, the broadcasted campaigns caused a stir among the population due to the seriousness of the images shown. In some cases, the immediate consequences of a traffic crash were shown, as in “You will pay for your recklessness” (1992). In other cases, the long-term consequences for the direct victims and/or their families were shown. This occurs in the real testimonies in the campaign “The story of … " (1994) or in “You are not the only one who pays for your recklessness” (1995), which recount the terrible situation in which the different family members of the victim of the traffic crash are left.

The advertisements present the consequences of traffic crashes in a raw, direct manner, without softening anything, seeking to trigger negative emotions in the spectators. This strategy aims to predispose people to make significant cognitive changes. In this way, the focus consists in taking advantage of the fear of drivers to achieve a true change in attitude and behaviour on the road, which would have a potential impact on the reduction in the number of injuries and deaths as a result of road traffic crashes. Therefore, the tone of the spots contributes to generating a genuine fear in drivers, which could influence their behaviour significantly, forcing them to respect road rules and adopt necessary precaution measures to avoid road tragedies.

This change in the communicative strategy, in a complementary way to the implementation of other preventative actions, contributed to the reduction in the number of victims at the beginning of the 1990s (Fig. 2). Nonetheless, this trend was established a few years later, which motivated a new change in communication resources and the way of directing themselves at an audience.

#### 4.1.4 Multivariate period (1998–2010)

This stage runs from 1998 to 2010 and is characterised by a mix of campaign styles, comprising informative, emotional, and impactful ads. The characteristic tools of the soft and hard periods are interspersed and interact with a certain ambiguity, which makes a clearer classification difficult. There is also an evolution at the aesthetic level, making use of higher quality technical tools, and even with the incursion of cinematographic resources. In this way, there is a succession of awareness campaigns that employ different techniques with the aim of capturing the spectator's attention and not allowing them to become desensitised (Table 4).

**Table 4:** Characteristics of national traffic campaigns between 1998 and 2010.

Campaign	Year	Theme	Technique
Live	1998	Alcohol, seat belts, speeding, fatigue	Informative
Teaches road safety education. Teaches how to live	1999	Helmet, child restraint system, drugs	Informative
Save yourself a hard time	1999	Alcohol	Metaphorical
They couldn't help it. You can	2000	Travel	Comparative
Rest every 200 km, you can avoid it.	2001	Fatigue	Informative
Answer B could always have been avoided	2001	Helmet, seatbelt, speeding, alcohol	Impact
Live and let live	2002	Travel	Informative
The first rule of pedestrian traffic is common sense.	2002	Pedestrian crossings	Informative
Embrace life	2003	Seat belt	Impact
Before making excuses, think about it	2004	Safety distance, helmet, speeding, seat belt	Impact
Why wasn't I wearing my seat belt?	2005	Seat belt	Emotional
Not a single drop of alcohol at the wheel	2005	Alcohol	Emotional
We will continue	2006	Travel	Informative
Do you think you will die on the road?	2006	Travel	Informative
Points system: How does it feel to be alive?	2006	Points-based driver's license	Informative
There are many reasons to …	2007	Risk factors	Emotional
Where do I go with a turkey?	2007	Driving license	Humour
In the car, you can live it all or lose it all.	2007	Fatigue	Emotional
Whatever age you are, drive with zero alcohol.	2008	Alcohol	Emotional
You can change reality	2008	Vulnerable users	Emotional
Hopefully you will be very distracted this summer, but when driving, be careful!	2008	Distractions	Emotional
We all count, we all discount	2008	Travel	Informative
We all know how it's done	2009	Travel	Emotional
Always wear the case, even in the city	2009	Helmet	Informative
In case of emergency	2009	Emergency	Informative
Seat belts, no excuses	2010	Seat belt	Informative
We all know how to avoid an accident, why don't we do it?	2010	Travel	Emotional
Enjoy the road, but do it safely	2011	Travel	Emotional
What we want is for you to arrive	2011	Alcohol	Emotional

The most represented themes are maintained and include distractions, which increased among drivers during those years due to the progress of technology and the appearance of the first mobile phones [49]. In fact, there are even campaigns specifically focused on distractions, such as “I hope you get distracted a lot this summer, but be careful when driving!” (2008). In addition, it is worth mentioning the introduction of the driver's licence points system. For this moment, this was a new preventive measure approved by the authorities and was the core focus of the traffic campaigns developed during 2006.

With the arrival of the first Strategic Road Safety Plan launched in 2005, the campaigns redirect the message once again. Thus, the vigilant and punitive approach that sometimes still exists, disappears completely to give way to a vision that includes the spectator as an active entity in crash prevention. Users are no longer educated because it is taken for granted that they are aware of the rules and their importance, but rather their role on the road through ads with a reflective tone is emphasised.

Some useful (or, at least, very informative) examples of this way to present road safety-related messages include “Do you think you will die on the road?” (2006), “We all know how it's done” (2009), or “We all know how to avoid an accident, why don't we do it?” (2010). In them, the viewer is urged to visualise himself as a possible victim of a traffic crash, to consider the reasons why they do not drive properly, and to change this attitude.

#### 4.1.5 The Last Ten Years (2011–2021)

The beginning of this stage is linked to the Strategic Road Safety Plan 2011–2020. In this period, the advertisements maintain the variety of communication strategies and resources used previously (Table 5). The most repeated themes each year are speeding, alcohol consumption and distractions. On the other hand, although they have not completely disappeared from the screens, there has been a reduction in campaigns specifically focused on the use of seat belts and helmets. This is because awareness of these risk factors has increased, and constant reminders are not so necessary. In any case, the topics are still very varied, with elements such as sustainable transport and journeys also appearing.

**Table 5:** Characteristics of national traffic campaigns between 2011 and 2021.

Campaign	Year	Theme	Technique
Someday accidents will be a thing of the past	2011	Travel	Informative
At your side we all go	2012	Travel	Informative
On the road, as in life, we are all connected.	2012	Commuting	Informative
Safety on the job starts when you leave the house	2012	In itinere displacements	News
You have to be where you need to be	2012	Distractions	Humour
Summer is full of life, and lives, respect them.	2013	Excessive speed	Impact
If you don't fasten your child restraint system, it's as if you're not wearing one at all.	2013	Child restraint systems	Impact
The best stories of summer are the ones you can tell	2013	Travel	Emotional
Thank you for arriving	2014	Travel	Informative
At the wheel 99% of the attention is not enough	2014	Distractions	Humour
You don't have to be on the road to cause an accident	2014	In itinere travel	Impact
Take your drugs out of circulation	2014	Drugs	Emotional
If you use drugs the victim is not only you	2014	Alcohol and drugs	Emotional
Move with conscience	2015	Sustainable transportation	Humour
The most expensive objects in the world	2015	Drugs and distractions	Impact
Your life's journey: Your life is a journey, our job is to help you protect it.	2016	Travel	Metaphorical
The 2%	2016	Alcohol	Metaphorical
Testimonials	2017	Distractions	Emotional
Magic: If you look at your cell phone once in a while behind the wheel, you can only see the road once in a while.	2017	Distractions: cell phone	Impact
It's clear you know how to pass. In the car, ignore your cell phone	2017	Distractions: cell phone	Informative
In a traffic accident, who would you rather be: who lives or who dies?	2018	Speeding, distractions and alcohol	Impact
The glass man	2018	Helmet	Metaphorical
Top vacational	2019	Speeding, distractions and alcohol	Dark humour
This country can't take any more deaths	2020	Travel	Metaphorical
After what we've been through to get to this summer, don't mess it up	2021	Travel	Humour
Not wearing your seat belt seems to be a thing of the past	2021	Seat belts	Impact

A distinctive nuance in this stage is the great importance placed on people. Obviously, traffic campaigns have always been aimed at road users, but in recent years there has been an increased effort to convey the importance of each human life that travels on the road and whose death is avoidable. In fact, one of the most repeated concepts in the campaigns of this period is “life”, appearing in “On the road, as in life, we are all connected” (2012), “Summer is full of life, and lives, respect them” (2013), “The journey of your life: Your life is a journey, our job is to help you protect it” (2016), “In a traffic accident, who do you prefer to be who lives or who dies? (2018) or “This country can't take any more deaths” (2020).

The reflective nature of many of the ads is maintained, and a high emotional impact is achieved without necessarily using images of great visual impact. In fact, the number of campaigns that use irony and humour increases and still achieve this objective. This is the case of “Top Vacational” (2018), where a sympathetic reporter reports on possible tourist destinations you can go to after a traffic accident, i.e. prison, hospital and the cemetery. The holiday campaign, conducted after the coronavirus crisis, is also not visually aggressive at all, but it has a shattering slogan: “After what we have gone through to get to this summer, don't spoil it” (2021). Thus, the Directorate-General for Traffic is developing heterogeneous campaigns with a direct message to the individual.

### 4.2 Evolution of communication strategies used in Spanish traffic campaigns

Once the five time periods had been identified, and their differing characteristics regarding content and communicative strategies offered, the descriptive quantitative analysis outcomes are presented.

Firstly, the marketing resources and techniques have been grouped in three big categories. In this way, the evolution of the merely informative campaigns, the largely emotional and impactful ones, and the humorous ones are separated. Thus, regarding the distinction or initial categorisation made in the frequency analysis (see section 3.3), the informative advertisements comprise those that have used animations, news, graphics and metaphors. That is to say, all advertisements that do not employ emotion to transmit their information. The emotional advertisements include all those spots that directly appeal to the audience with an emotive tone, including those that do not use aggressive (or impactful) images, as well as those that do. The third category corresponds to humorous advertisements, including spots of diverse nature that use sarcasm, irony, exaggeration, or comical scenes and/or dialogue, among other communicative techniques.

**Fig. 3:**
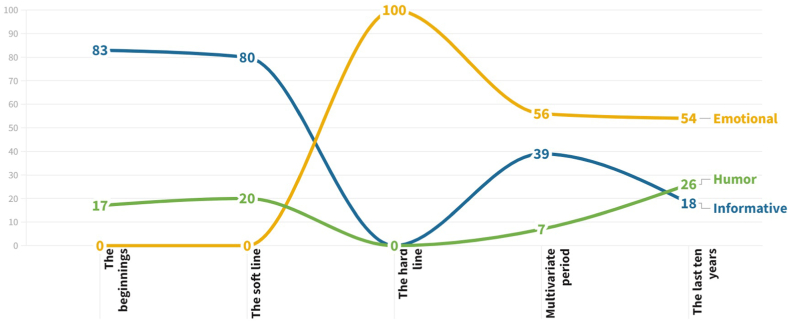
Evolution in communication techniques in Spanish traffic campaigns.

Thus, Fig. 3 represents the evolution of the communicative techniques used in traffic campaigns in Spain according to their techniques and the emotional focus. Initially, informative advertisements were largely used. However, especially from the period of “The Hard Line”, more impactful techniques were used that appealed to the audience's emotions. Consequently, in recent years, numerous campaigns have been carried out in which different communication strategies are interwoven.

The presence of humour is highlighted in almost all periods. This phenomenon may be surprising and shocking, since it is a technique that appears to contradict the gravity and severity of the problem of road accidents. Nonetheless, the effectiveness of humour is supported by various investigations that highlight the role of surprise as a key factor in capturing an audience's attention, as well as the understanding of images and messages, contributing to the effectiveness of the advertisement [50]. Furthermore, the importance of choosing the right humorous resource was highlighted, as the most effective ones were “absurd” and “comparisons” and/or “exaggerations”. The reason was that they were simpler for an audience to understand compared to satire and irony, which can distort the de-codification or interpretation of the message [51].

In any case, the current variability in terms of communicative strategy is considered by the evidence to be the most effective manner to raise audience awareness. The heterogeneity in the form of communicating contributes to the no sensitisation of the spectator and, therefore, to the higher levels of attention and retention of information [52].

**Fig. 4:**
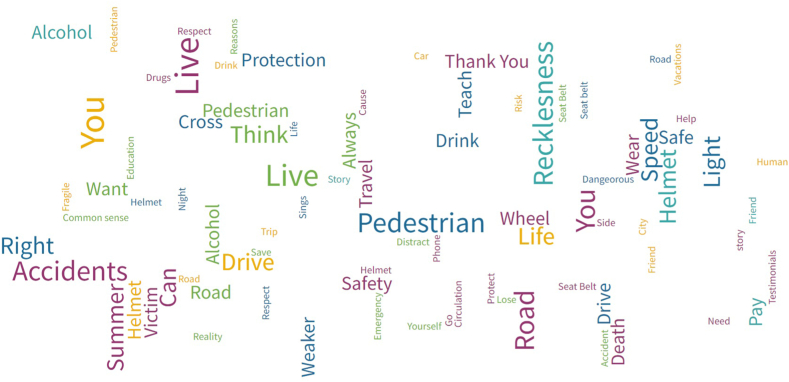
Representation of the most used words in slogans of Spanish traffic campaigns.

Notes: The size of the words depends on the number of occasions in which it was repeated in slogans, meaning the bigger the word, the greater number of times it appeared in the corresponding time period.

Secondly, a graphic representation of the frequency of use of the main words in the selected campaign's slogans has been created. Fig. 4 shows the most used words in the awareness campaign slogans. Five different colours are used, which correspond to the five time periods identified (dark blue = The Beginnings; yellow = The “Soft” line; light blue = The “Hard” line; green = Multivariate Period; purple = The Last Ten years). So, there may be repeated words since they can be used in different time periods, in which case they are represented in their corresponding colours.

Consistency can be observed between the most used strategies in each time period and the words used. In “The Beginnings”, nouns such as pedestrian, speed, right and teach are highlighted. These words present educational connotations, transmitting the descriptive and informative tone employed in this period. Words intended to alert the spectator are not highlighted, but solely educate them about the rules and safe behaviour that should be maintained when driving.

“The Soft Line” period starts to mention words related to the user, presenting specific risk factors and the consequences of not following road rules. Thus, concepts such as you, drive, life, which suppose a change in trends in comparison to the previous time frame are highlighted. In contrast, it is important to outline the hardness of the words used during the “Hard Line” period, such as recklessness or pay. So, it is particularly insistent that users can pay the price for their risky behaviour behind the wheel, and they also introduce specific risk factors such as helmet or alcohol.

Finally, in the last time periods (“The Multivariate Period” and “The Last Ten Years”), the campaigns are directed at the user, underlining words such as *you*, *thank you*, *victims* or *accidents*. Individual responsibility in insinuated in verbs like live, think or drive. The spectator already knows what safe behaviour is, thus the main cause of traffic crashes is shown as the human factor. They try to transmit that reducing the problem of road “accidents” is in the hands of the drives and other road users, insisting on the importance of the individual actions and behaviour of users to avoid crashes.

### 4.3 Contribution of communication campaigns in the reduction of traffic crashes: a link in the chain of preventive measures

Mass media are direct channels of communication with the population, with a considerably great reach [53]. For this reason, advertising campaigns promoting safe driving have been indispensable over the years [54]. Although it is true that the effectiveness of campaigns in isolation has not been frequently evaluated [55], it has been found that traffic advertisements have been claimed to contribute to crash reduction when combined with other measures, such as regulations and sanctions, or road safety training [56]. In this sense, campaigns serve as an informative and educational tool regarding all actions developed in each country in the traffic and mobility sector [57,58].

Following this idea, not all communication strategies employed in traffic campaigns have proved to be equally effective [59]. In this sense, one should first define what is meant by the effectiveness of a traffic ad. This is important because when we analyse the effect of a consumer product advertisement on viewers, variables such as campaign recall, brand awareness or predisposition to buy are considered [60]. However, before a campaign is framed within social marketing, the most important result is the change in attitude and behaviour of the citizen towards more prosocial behaviours, which, ultimately, have an impact on both personal wellbeing and that of society as a whole [[61], [62]]. For this reason, a parallel will be drawn between the road crash figures in Spain and the characteristics of the traffic campaigns between 1960 and 2021, without ignoring the role of other actions and preventive measures that have been developed during these years.

The first traffic advertisements in Spain had a marked informative character (Segura-García, 2015). They were mainly intended to educate the population about the new regulations that were being implemented in the country. This was a period where the increase in the number of cars in circulation has been claimed to have a positive correlation with the growing trend of traffic crashes [63]. The communication strategy carried out in the advertisements, as well as the sanctions applied during this period, did not manage to reverse this trend. In this sense, and as other studies show, it is complicated to achieve a behavioural change through merely informative spots [64]. Advertisements with clear, persuasive, and repetitive slogans played a role in the transmission and recall of norms, but recalling a slogan does not necessarily have to be accompanied by a real change in behaviour [65,66]. In fact, the profile of the driver is a key element to understand why for some people, this type of advertisement is sufficient, but for other users, it is not. Drivers with a low-risk profile adapt their behaviour according to the norms exposed by ads [67]. However, users who habitually engage in the most dangerous behaviours are more likely to resist behavioural change [68].

With this idea in mind, the communication strategies used in these spots were modified to try to capture the attention of citizens and convince them of the importance of proper driving, regardless of their profile as a driver [69]. Thus, one enters the “soft-line” where messages no longer seek to educate on behaviour since it is assumed that users are knowledgeable about the regulations. In this way, the consequences of inappropriate behaviour begin to be shown. Even so, the trend in the number of deaths continues to grow, so the measures adopted have not been sufficient.

A potentially reasonable explanation could be that, although negative consequences are commonly exposed in these campaigns, the tone of the spots remains ‘friendly’, as suggested by Martínez-Rodrigo & Segura-García (2013) [70] in a previous empirical study. That is, the images are of low impact, making use of animations and famous characters that can help to recall the message, but as already commented, they have been argued to be not very effective for behavioural change in recent empirical studies [35,64].

#### 4.3.1 1990s and on: hard-line as the “cornerstone” of road safety persuasion

This is how we enter the 1990s and the so-called “hard-line” in which the consequences of casualties are depicted with rawness and violence. The visual and emotional impact of the images is high because realistic scenes of severe crash events and the repercussions they have on the victims and their environment are exposed. This type of advertisement has proved to be much more effective than the ones used so far [71]. In fact, an abrupt change in crash rates and fatalities was observed at the beginning of this period. Numerous studies emphasise that the emotion of the audiovisual piece is a key variable in behavioural change [72]. In addition, it should be emphasised that at the beginning of this period, changes were made in the regulations that also favored the reduction observed in the rates of traffic crash-related fatalities. As a result, substantial modifications were made to many preventive measures that led to a change in the road crash trend.

In relation to the role of the hard-hitting campaigns disseminated during this period, these advertisements achieve a generation of empathy in the spectator by making them identify with the protagonists, forcing them to reflect on what actions they can take to avoid similar situations. Furthermore, the use of fear as a stimulus in the user undoubtedly constitutes a powerful motivator to encourage a change in attitude and behaviour in the road environment [73]. However, casualty rates increased again during the late 90s despite this hard line of spots. Why does this happen? The main reason is that the viewer becomes desensitised to the high levels of rawness in the images. This supports the asumption that violent scenes do not generate the same impact if they are broadcast over a prolonged period of time, but neither can they be completely eliminated because they have proved to be effective, as suggested by studies such as Lewis et al. (2019) [74] and Slater et al. (2012) [75].

At this point, the multivariate stage begins, where the most aggressive campaigns are complemented with other types of resources in which emotion and information are maintained, but are not so visually impactful [76]. According to several studies, this is the ideal dynamic. Thus, spots with a high level of hardness are developed after a prolonged period of mild and moderate impact advertising [77]. In this way, the multivariate period is characterised by making use of different narrative resources with sensitive, creative, and even humorous cut campaigns, and still making the campaign effective [78,79]. Another relevant element that also appears in this era is the number and limited duration of ads. It is known that communication campaigns are usually more effective when they are of short duration and when they are linked to a specific measure [36,80].

Thus, most large campaigns carried out in Spain in these years can be understood as seasonal. That is, the preventive messages of the spots are usually broadcast during holiday periods when a greater number of trips are conducted, and serve as a support for sanctions and increased police surveillance. This combination has a positive impact on the effectiveness of the measures and achieves the purpose of reducing road casualties [56].

### 4.4 Balance and current status of traffic campaigns in Spain

Communication campaigns in the traffic and road safety sector are one of the most widely used preventive measures worldwide [16]. The last few years of their development have followed the dynamics of the use of varied resources, among which are interspersed spots of an emotional, informative, and impactful nature. But it is worth noting the particularly reflective nature of the ads during this period.

The results of this study have shown how (at least in the Spanish case) the aim of these actions seems no longer to be to instil fear among viewers, but rather to play with more psychological aspects different to primary –and negative– emotions. Not only is a quick change sought, but a step further is also taken by putting lots of emphasis on the value of life, and inviting the user to become aware and rethink the way they circulate. This deep reflection can achieve more lasting changes in citizens because it allows them to modify their way of thinking and not just their imminent behaviour [81]. This allows playing with multiple resources and marketing techniques, making use of metaphors, comparisons, dark humour, or realism, among others. The diversity in the content of advertisements surprises spectators when they are presented with a spot, since they do not become accustomed to a specific style or theme. This situation generates a greater impact, since they allow the spectators' attention and interested to be maintained, which allows the advertisement's message to be transmitted more effectively. The variability of content also avoids the saturation and waste of communicative strategies, avoiding the desensitisation of the audience [52].

### 4.5 Limitations of the study

It is important to bear in mind some limitations of the current investigation to correctly interpret the results. Firstly, an analysis of the content of traffic communication campaigns broadcast in Spain was carried out. Not all the advertisements have been analysed, largely due to questions of availability, volume of data, and access to information. Consequently, selection bias may have occurred as there is a possibility that the selected advertisements do not fully represent the spectrum of publicity campaigns [82]. In any case, to minimise this bias and to ensure that the selection process was systematic and replicable, specific inclusion and exclusion criteria agreed by the authors were established.

Additionally, we consider that the 104 selected campaigns represent a sufficient amount of advertisements in each time period to be able to establish valid conclusions. Secondly, the categorisation of the advertisements may have been seen as biased by subjective interpretation of the data and content. To reduce this bias, the four authors conducted an independent evaluation and analysis, which provides consistency and validity to the categorisations [35,83].

Finally, it is worth highlighting that at no point did the authors state that the selected communication campaigns had been evaluated in relation to their efficiency in changing behaviour. The traffic advertisements are not applied in an isolated way, but rather act as a complement to other preventative measures, thus meaning that they can not be evaluated without considering the social context and other actions developed by the responsible organisations during each time period [84]. In this sense, the present study carries out a first approximation of the potential contributions of spots in the reduction of road accidents, but does not establish direct relationships of causality between advertisements and death rates.

## 5 Conclusions

The first key outcome of this study is endorsing the assumption that topics, techniques and communication strategies applied to the field of road safety have evolved over the years with the aim of improving their effectiveness.

In the Spanish case, several time periods can be differentiated where spots present certain common characteristics. In the first stages, informative and educational spots predominated and later, emotional and impactful scenes were introduced. The next step was to move on to the line which has been maintained to the present day, in which various resources and marketing techniques are interspersed to avoid desensitising the viewer.

This trend has proved to be the most appropriate, achieving good results, which are still increased if complemented with other preventive measures. Even so, it is necessary to continue evaluating traffic communication campaigns to assess the effectiveness of the tools used and thus, contribute to the improvement of future advertisements and their policy-related issues in this area.

## Funding statement

This study has been funded (Grant 20170475) and supported by the INTRANT (National Institute of Transit and Land Transportation) and the OPSEVI (its Permanent Observatory in Road Safety), public agencies of the Dominican Republic government and was supported by the research grant ACIF/2020/035 (MF) from “10.13039/501100003359Generalitat Valenciana”. The funders had no role in study design, data collection and analysis, decision to publish, or preparation of the manuscript.

## Declaration of competing interest

The authors declare that they have no known competing financial interests or personal relationships that could have appeared to influence the work reported in this paper.

## Figures and Tables

**Table:** 

Campaign	Year	Description of traffic campaign
Respect the signs (Respete las señales)	1960	A car drives without respecting signs, and almost has an accident.
One more drink can mean one less life (Una copa de más puede ser una vida de menos)	1961	Drawings of a vehicle “filling” with wine that an ambulance comes to rescue.
Overtaking is dangerous (Los adelantamientos son peligrosos)	1962	Drawings explaining how to overtake properly.
Drive at a safe speed (Circule an una velocidad prudente)	1962	Drawings showing the differences in braking depending on the speed of the car.
Look first, then cross (Primero mirar, después cruzar)	1963	Real drivers, motorcyclists and pedestrians crossing the road with caution.
Pedestrian! Always cross with a green light (Patón, cruce siempre con luz verde)	1964	Drawings teaching pedestrians how to cross the road.
Wear … Helmets (Usen … casco)	1964	Drawings of motorcyclists in different crash scenarios.
(Attention to speed) Atención a la velocidad	1965	Images of vehicles.
Cyclists on the right one after the other (Ciclistas por la derecha uno tras otro	1965	A policeman shows cyclists where to cross the street.
Keep on the right (Circule por la derecha)	1965	Different vehicles driving on the right side of the road.
Check your lights (Revisión de luces)	1965	Incorrect use of car lights dazzle other drivers
Check your lights (Revise sus luces)	1967	Images of vehicles with lights on.
Weaker ones need more protection (Los más débiles necesitan mayor protección)	1967	A father teaching his children where to play safely, avoiding cars, teaching them how to protect themselves.
Weaker ones need more protection (Los más débiles necesitan mayor protección)	1967	Series of advertisements with pictures of small animals protected by larger ones, simulating the extra protection for children.
Don't be like him (No sea como él)	1969	A picture of a family dressed in funeral dress because one of their family members has died.
Teach him to ride well (Enséñele an andar bien)	1969	Drawings of a family teaching a small child how to cross the road.
Pedestrian, on the road always keep on the left side of the road (Peatón, en carretera circule siempre por la izquierda)	1973	Drawings of a pedestrian explaining that he must keep the left to see the vehicles.
Pedestrians also have their rights (El peatón también tiene sus derechos)	1973	Drawings of a driver, reminding of the vulnerability of pedestrians.
Knowing how to walk is not enough, teach him how to drive (Saber andar no basta, enséñele a circular)	1973	Children running out of school and into the road where vehicles are.
Driving at night (Circulación de noche)	1973	Drawing explains to pedestrians how to be seen on the road at night.
For your safety, use seat belts (Por su seguridad, utilice el cinturón)	1973	Drawings show the consequences of a crash with and without a seatbelt.
Give them time (Dele tiempo)	1976	A vehicle starts as soon as the green light changes, almost hitting a pedestrian.
Always with one glass less (Siempre con una copa de menos)	1978	Images of a driver refusing a glass of wine.
When in doubt, do not go ahead (Si duda, no adelante)	1978	A driver explains how to overtake correctly.
Helmet, the only compulsory item of clothing (Casco, única prenda obligatoria)	1981	A couple in swimming trunks get on a motorcycle with a helmet on.
Keep an eye on your tires (Vigile sus neumáticos)	1981	Drawings of a driver checking his tires.
City in chaos (Ciudad sumida en el caos)	1982	Drawings of cars driving in a city.
Vacations are for living (Las vacaciones son para vivir)	1983	A couple in a bathing suit on a motorcycle with a helmet.
Thank you for driving well (Gracias por conducir bien)	1984	Different children looking at the camera thanking the drivers for their behaviour.
Be prepared for winter driving (Prepárese para conducir en invierno)	1984	A toy car drives around in different weather conditions.
You are the most important part (Usted es la pieza más importante)	1984	Voice-over explains that although vehicles have improved their conditions, there are more and more accidents.
If you drink, don't drive (Si bebes no conduzcas)	1985	Stevie Wonder reminds “If you drink, don't drive".
In every car there are human lives (En cada coche hay vidas humanas)	1985	Car seats with people ride on the road.
In a car, you are the most fragile thing (En un coche, usted es lo más frágil)	1986	A car with glass passengers has an accident, shattering them into pieces.
Many lives depend on you (Muchas vidas dependen de usted)	1987	Drivers of public transport (plane, train, ship) appear, and later a car driver, and many lives depend on them all.
Using the best car insurance is caution (Utilice el mejor seguro del automóvil es la precaución)	1988	A mug of beer is thrown until it crashes against a bar.
On the road, don't go out like bullets (En carretera, no salga como las balas)	1988	A bullet man is shot out of a cannon.
A friend tells you (Te lo dice un amigo)	1989	A drawing of a squirrel is superimposed on real scenes explaining the dangers of breaking the rules.
Pedestrian! There is nothing as fragile as your body (Peatón, no hay nada tan frágil como tu cuerpo)	1990	A drawing of a pedestrian gets run over.
Don't make an adventure out of your trip (No haga de su viaje una aventura)	1990	Indiana Jones explains that reckless driving should only be done in fiction.
A champion's word. Don't take risks. You'll come out on top (Palabra de campeón. No arriesgue. Saldrá ganando)	1991	Carlos Sáinz and Luis Moya explain the importance of complying with road rules.
Get it into your head (Métetelo en la cabeza)	1991	An open walnut simulates the consequences for the head in a crash without a helmet.
Fasten your seat belt (Abróchese el cinturón)	1991	Different belts appear, to conclude that there is one that saves your life.
Recklessness is paid for more and more each time (Las imprudencias se pagan cada vez más)	1992	Scenes right after an accident with medical staff and emergency personnel.
In the end, recklessness is paid for (Al final, las imprudencias se pagan)	1993	Images of injured people interspersed with accident scenes.
The story of … (La historia de …)	1994	Traffic accident victims tell their testimonies.
You're not the only one who pays for the recklessness (Las imprudencias no solo las pagas tú)	1995	Images of relatives of victims of traffic accidents and the consequences for them.
The solution is in your hands (La solución está en tus manos)	1996	The voiceover asks about the symptoms of suffering a respiratory arrest/polytraumatism, and the answer is people talking about the imprudence they commit that can lead to a traffic accident.
Show your friendship, do not allow recklessness (Demuestra tu amistad, no permitas imprudencias)	1997	Scenes of people wanting to commit recklessness to which a friend tells them seriously that they can do it. They emphasise not to commit such acts.
Live! (Vive)	1998	Several people directly address the viewer to tell him that they promise to comply with road rules because they want to continue living.
Teache road safety education! Teach how to live! (Enseña educación vial. Enseña a vivir)	1999	Series of advertisements with routine scenes where someone makes a preventive recommendation (unrelated to road safety education), to emphasise the importance of teaching people to protect themselves while driving.
Save yourself a bad drink (Ahórrate un mal trago)	1999	A car crashes into a big glass of alcohol.
They couldn't help it. You can (Ellos no pudieron evitarlo. Tú sí puedes)	2000	Images of natural catastrophes with hundreds of deaths, contrasting scenes of deaths in traffic accidents that could have been avoided.
Rest every 200 km, you can avoid it. (Descansa cada 200 km, tú si puedes evitarlo)	2001	Apparently there are images of an accident involving a man, but it is later discovered that he was simply resting.
Answer B could always have been avoided (La respuesta B siempre se podía haber evitado)	2001	Different scenes of injured and deceased people in which two options are given for what happened. One related to birth problems and the other due to a traffic accident that could have been avoided.
Live and let live (Vive y deja vivir)	2002	Scenes that could have ended in an accident are avoided because the driver keeps his cool.
The first rule of pedestrian traffic is common sense (La primera norma de circulación del peatón es el sentido común)	2002	Pedestrians are urged to make sure vehicles stop and drivers are urged to stop at zebra crossings to avoid an accident.
Embrace life (Abróchate a la vida)	2003	Crashes without a seat belt are compared to impacts in other contexts (falling off a building, being hit with a hammer, etc.).
Before making excuses, think about it (Antes de poner excusas piénsalo)	2004	Situations where non-compliance with regulations leads to an accident that can be avoided with appropriate actions.
Why wasn't I wearing my seat belt? (¿Por qué no llevaba puesto el cinturón?)	2005	A doctor asks patient after patient, why wasn't he wearing his seat belt?
Not a single drop of alcohol at the wheel (Al volante ni una sola gota de alcohol)	2005	A man at a party while the voiceover narrates his last times because he will have an accident caused by the alcohol ingested.
We will continue (Seguiremos)	2006	Scenes in which everything the DGT will do is told (more campaigns, better infrastructure, etc.), but without the users, nothing is achieved.
Do you think you will die on the road? (¿Cree usted que morirá en la carretera?)	2006	An operator asks if he thinks he will die in a traffic accident, to which the man answers no, but it is estimated that 100 people will do so during Easter week.
Points system: How does it feel to be alive? (Carnet por puntos: ¿Qué se siente al seguir vivo?)	2006	Voiceover states that more than 60,000 people have lost points but that there have been 150 fewer road deaths.
There are many reasons to … (Hay muchas razones para …)	2007	Several commercials were made, each one focused on a risk factor (speeding, seat belts, alcohol, etc), all of them with a man looking at the camera and asking the viewer to find a reason to avoid them.
Where do I go with a turkey? (¿A dónde voy yo con un pavo?)	2007	A boy wakes up with a turkey and doesn't know what to do with it until he hears that for a turkey a day he can get his driver's license.
In the car, you can live it all or lose it all. (En el coche puedes vivirlo todo o perderlo todo)	2007	Different scenes of situations lived in a car, to visualise a crash due to falling asleep at the wheel and losing his life.
Whatever age you are, drive with zero alcohol. (Tengas la edad que tengas, conduce con cero alcohol)	2008	A man looking at the camera tells the viewer that he can get caught or crash if he drinks while driving, showing that no one is immune.
You can change the reality (Tu puedes cambiar la realidad)	2008	A man looking into the camera says that he killed a motorist because he didn't see him, then says it's not serious in his case because he's an actor.
Hopefully you will be very distracted this summer, but when driving, be careful! (Ojalá te distraigas mucho este verano, pero al volante ¡atento!)	2008	A man examines the wreckage of a traffic accident and establishes causality between each element and actions that cause distraction (cell phone, looking at glove box, etc.)
We all count, we all discount (Todos contamos, todos descontamos)	2008	Safe driving will reduce the number of deaths due to accidents.
We all know how it's done (Todos sabemos cómo se hace)	2009	The course exposes common sense scenes in reference to traffic situations, to conclude: if we know that by respecting the traffic regulations there will be fewer victims, why not do it?
Always wear the helmet, even in the city (Ponte el caso siempre, también en ciudad)	2009	Situations where a motorcycle accident may occur are presented, hence the importance of always wearing a helmet.
In case of emergency (En caso de emergencia)	2009	The importance of marking with an “AA” in front of the person we want to be contacted in case of emergency, speeding up the work of those in charge.
Seat belt without excuses (Cinturón de seguridad sin excusas)	2010	Situations where people do not wear seat belts and should wear them (as a passenger in a cab, on short journeys, etc.).
We all know how to avoid an accident, so why don't we do it? (Todos sabemos cómo evitar un accidente, ¿por qué no lo hacemos?)	2010	Reactions of different people who receive the news by phone that a family member has had a fatal accident.
Enjoy the road, but do it safely (Disfruta del camino, pero hazlo de forma segura)	2011	Scenes of road users for the purpose of safe travel.
What we want is for you to get there (Lo que queremos es que llegues)	2011	People are thanked for having prevented someone who had been drinking from driving.
One day accidents will be a thing of the past (Algún día los accidentes serán cosa del pasado)	2011	A group of people from the future look at the remains of a traffic accident in a similar way as if they were looking at archaeological remains.
By your side we all go (A tu lado vamos todos)	2012	Scenes of different families welcoming a driver showing the importance of driving carefully to reach the destination.
On the road, as in life, we're all connected (En la carretera, como en la vida, todos estamos conectados)	2012	Scenes of road users performing appropriate actions with respect for other road users.
Safety on the job starts when you leave the house (La seguridad en el trabajo comienza al salir de casa)	2012	Scenes in different work environments where workers say “I just go from home to work and from work to home".
You have to be where you need to be (Hay que estar a lo que hay que estar)	2012	Leo Harlem does a series of monologues about distractions of road users.
Summer is full of life, and lives, respect them (El verano está lleno de vida, y de vidas, respétalas)	2013	A family is brushing their teeth when a sudden crash is heard and they collapse in their bathroom.
If you don't fasten your child restraint system, it's as if you're not wearing one at all. (Si no abrochas el sistema de retención infantil es como si no lo llevara)	2013	A girl is thrown from a car for not properly wearing a child restraint system.
The best stories of summer are the ones you can tell (Las mejores historias del verano son las que se pueden contra)	2013	A series of advertisements about people's vacations that would not have taken place if they had been involved in an accident.
Thank you for arriving (Gracias por llegar)	2014	People arriving in a village at Christmas.
At the wheel 99% of the attention is not enough (Al volante el 99% de la atención no es suficiente)	2014	Different scenes (operating room, air traffic controller) where it is evident that the attention must be completely focused on the action, just as it must be when driving.
You don't have to be on the road to cause an accident (No es necesario estar en la carretera para provocar un accidente)	2014	Actions of the driver's environment (the boss making him leave later, the partner calling him while driving, a pedestrian crossing where he shouldn't) can lead to an accident with many people involved.
Take your drugs out of circulation (Saca tus drogas de la circulación)	2014	A man is driving on drugs and suddenly cannot get out of his vehicle. There are thousands like him, and it is eventually discovered that it is because they have been killed in an accident.
If you use drugs the victim is not only you (Si consumes drogas la víctima no eres solo tu)	2014	A man is shown to die as a result of a driver's drug use, without explicit images.
Move with conscience (Muévete con conciencia)	2015	It shows a man driving to work, where every day he gets angry because of the time he wastes in traffic jams, parking, among other things. Showing the option of cycling and public transport as a less stressful alternative.
The most expensive objects in the world (Los objetos más caro del mundo)	2015	Objects and actions such as a pair of glasses, a change of radio station or a joint can be the cause of an accident, with dire consequences for all involved.
Your life's journey: Your life is a journey, our job is to help you protect it. (El trayecto de tu vida: Tu vida es un trayecto, nuestro trabajo es ayudarte a protegerlo)	2016	People appear with their daily routines through the kilometres travelled during their lives.
The 2% (El 2 por ciento)	2016	Different elements appear that represent 2% of something, showing how little it represents of the total. But then it is said that this percentage means that 400 people die every year after an accident due to alcohol consumption while driving.
Testimonials (Testimoniales)	2017	Real testimonies from victims of traffic accidents.
Magic: If you look at your cell phone once in a while behind the wheel, you can only see the road once in a while. (Magia: Si al volante miras el móvil de vez en cuando, solo ves la carretera de vez en cuando)	2017	Vehicles are shown appearing and disappearing, until suddenly a woman is hit by a car, showing the information on the road that the driver loses if he looks at his cell phone.
It's clear you know how to pass. In the car, ignore your cell phone (Está claro que sabes pasar. En el coche, pasa del móvil)	2017	Scenes of a girl not paying attention to the comments of several people, and then not answering a call while driving.
In a traffic accident, who would you rather be: the one who lives or the one who dies? (En un accidente de tráfico, quién prefieres ser ¿quién vive o quién muere?)	2018	The negative consequences of a traffic accident are shown for the one who lives and the one who dies, taking into account personal, family and work aspects, among other things.
The glass man (El hombre de cristal)	2018	Image of a motorcyclist in glass shattering into pieces after a crash.
Top holidays (Top vacacional)	2019	Three possible destinations are shown after a traffic accident as holiday destinations: prison, hospital and a cemetery. In each case, their characteristics are presented, simulating a reporter's journalistic report.
This country can't take any more deaths (Este país no puede soportar más muertes)	2020	Skateboarder Javier Fernández skates on the Palacio de Hielo in Madrid, which was used as a morgue during the health pandemic derived from the COVID.
After what we've been through to get to this summer, don't mess it up (Después de lo que hemos pasado para llegar an este verano, no lo estropees)	2021	Irresponsible actions of different road users without shocking or aggressive images.
Not wearing your seat belt seems to be a thing of the past (No ponerse el cinturón parece cosa del pasado)	2021	Beginning with an advertisement from the 70's, to abruptly change to images of a deadly crash in the present day.
